# Progression of myocardial fibrosis by magnetic resonance imaging in patients with Duchenne and Becker muscular dystrophy

**DOI:** 10.1186/1532-429X-15-S1-P148

**Published:** 2013-01-30

**Authors:** Marly C Silva, Zilda M Meira, Juliana G Giannetti, Marco A Lara, Mariz Vainzof, Mayana Zatz, Roberto Kalil-Filho, Carlos E Rochitte

**Affiliations:** 1 Cardiology, Heart Institute - InCor, Sao Paulo, Brazil; 2 Federal University of Minas Gerais, Belo Horizonte, Brazil; 3 Human Genome Research Center, Bioscience Institute, University of São Paulo, São Paulo, Brazil

## Background

Duchenne (DMD) and Becker (BMD) muscular dystrophy (MD) are inherited X-linked disease characterized by progressive skeletal muscle degeneration and myocardial involvement and caused by a mutation on dystrophin gene (Xp21). Dystrophin is a sarcolemal protein that links the cytoskeleton to the basal lamina and is essential for maintenance of the muscular membrane integrity during muscular contraction. In a previous study, our group described for the first time, in a small group of 10 patients, that CMR can identify myocardial fibrosis (MF) and may be useful for detecting the early stages of cardiomyopathy in MD [1]. The evolution of the myocardial fibrosis over time in this group of patient is still unknown.

## Methods

We enrolled 76 pts, 70 pts with DMD and 6 BMD. Mean age was 13.1 ± 4.4 years. 74 patients underwent 2 CMR exams within a mean of 2.3 years, performed on a 1.5-T Siemens Avanto (Erlangen, Germany). Breath-hold LV short-axis and long-axis images were obtained by 2-pulse sequences at the same locations, allowing precise comparisons between LV function (gradient-echo sequence, steady-state free precession) and myocardial structure (inversion-recovery prepared gradient-echo sequence, 10 min after intravenous bolus or 0.2 mmol/kg gadolinium-based contrast), acquired with the following parameters, respectively: TR 2.0/ 9.0, TE 1.07/ 5.0; flip angle 69/50; cardiac phases 20/1; VPS 8/16 to 32; matrix 192 x 162 / 256 x 192; ST 8/8 mm; gap 2/2 and FOV 32 to 38/32 to 38 cm; TI none/150 to 390 ms; receiver bandwidth 930/150 Hz; number of excitations 1/1; acquisition every other heartbeat for MDE.

The images were analyzed by two experienced observers. End-systolic LV volume, end-diastolic LV volume and LV ejection fraction were measured by the Simpson method using Argus software, Siemens. For the MDE short axis images, we evaluated the MF mass per patient, using a 5 standard deviation thresholding technique, on a CMR42 software, version 3.4.2 (Circle Cardiovascular Imaging, Calgary, Alberta, Canada). Normal LVEF was defined as above 50%. Test of Wilcoxon was used for comparison of myocardial fibrosis between baseline and follow-up CMR studies.

## Results

Two patients died before the follow-up CMR. For all 74 patients, MF increased significantly from 11.3 ± 12.2 g on the baseline CMR to 15.9 ± 15.4 g, p<0.001. The number of segments with MF increased from 3.7 ± 3.3 to 5.1 ± 3.6, p<0.001. In a sub-group with LV dysfunction (n=11) MF increased from 26.9 ± 13.0 g to 35.5 ± 20.2 g, p=0.021. In a sub-group of patients with normal LVEF and no fibrosis (n=21) on the baseline CMR, MF progressed from 0 g to 1.5 ± 3.7 g, p=0.08.

## Conclusions

Myocardial fibrosis detected by CMR progressively increases in patients with Duchenne and Becker muscular dystrophy over a period of 2.3 years. The progression occurs not only in patients with more advanced cardiomyopathy and LV dysfunction, but also in patients with normal function and no myocardial fibrosis at the baseline CMR.

## Funding

Self Funding

**Figure 1:**
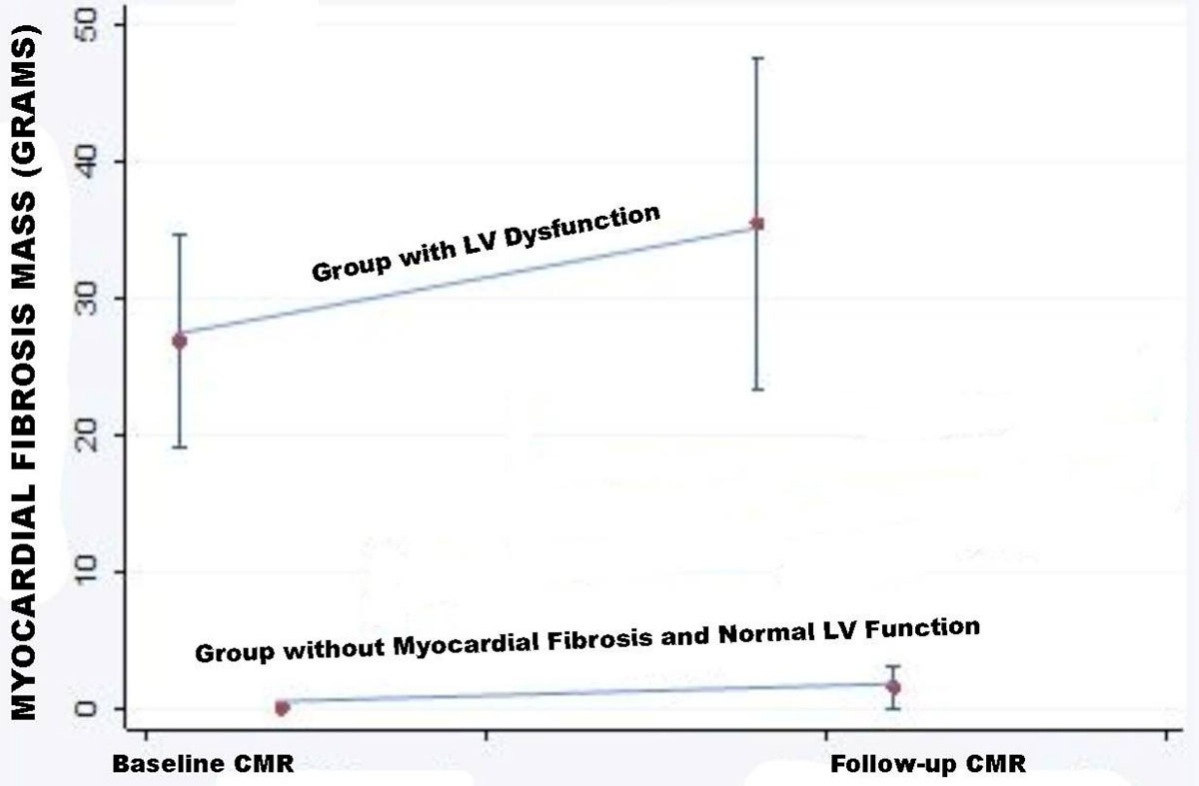
Progression of myocardial fibrosis by CMR between baseline and follow-up for groups with LV dysfunction and without myocardial fibrosis and normal LV function
